# Execution data logs of a supercomputer workload over its extended lifetime

**DOI:** 10.1016/j.dib.2019.105006

**Published:** 2019-12-16

**Authors:** Manuel Rodríguez-Pascual, Antonio J. Rubio-Montero, José A. Moríñigo, Rafael Mayo-García

**Affiliations:** ICT Division, CIEMAT, Avda. Complutense 40, 28840, Madrid, Spain

**Keywords:** Cluster computing, Computer science, HPC infrastructure, Parallel workload archive

## Abstract

The data of this research describes the logged usage of the Euler cluster located at CIEMAT (Centre for Energy, Environment, and Technology Research), spanning the period between November 2008 and March 2018. The Euler database is open access in Parallel Workload Archive format, available from the PWA repository [1] and Mendeley Data [2], allowing in this way a whole new bunch of research possibilities on computer science.

**Table:** Specifications Table

Subject	Computer Science
Specific subject area	Scientific Workload Traces of Supercomputers
Type of data	Data (human readable) file, Graphs
How data were acquired	Log files corresponding to the computing workload performed on Euler supercomputer, stored by PBS [4] over time, were compiled and re-formatted according to the Parallel Workload Archive standard. As an example of valuable statistical information that could be obtained from this dataset despite its size, a set of figures is built with Matplotlib [5,6] after processing the dataset with MariaDB [7].
Data format	Raw data (human-readable) file. Analyzed in the graphs.
Experimental Parameters for data collection	Euler is a Dell cluster located at CIEMAT. It is composed of 240 nodes with Dual Xeon 5450 quad-core, 144 nodes being 3.0 GHz and 96 nodes of 2.96 GHz, for a total of 1920 cores. Each of these nodes comes with 16 GB RAM, making a total of 3.8 TB. They are connected by two networks: a dual Gigabit Ethernet and a dual 4X DDR full non-blocking Infiniband connection. Network storage is managed with the Lustre FS, comprising a total of 120 TB of data. Euler cluster employs PBS [4] as the Local Resource Management System. MAUI [8] is employed as the external scheduler for PBS.
Description of data collection	Data (execution trace) were collected over time during the production period of Euler supercomputer. Collection starts since its initial deployment as the flagship computer at CIEMAT up to its replacement by newer hardware devoting it to auxiliary tasks. Start Time: Monday Nov 17 14:01:47 CET 2008. End Time: Sunday Dec 31 23:28:03 CET 2017 The execution trace comprises about nine million application executions (jobs) from more than 250 users over nine years of the supercomputer exploitation.
Data source location	Institution: CIEMAT City: Madrid Country: Spain
Data accessibility	Repository name: Parallel Workload Archive (PWA) Direct URL to data: http://www.cs.huji.ac.il/labs/parallel/workload/l_ciemat_euler/index.htmlhttps://dx.doi.org/10.17632/8bg3jkpgzp.1

**Table:** 

**Value of the Data** • Euler cluster database comprises a rich source of information regarding scientific users' behavior at computing. The statistical analysis of this database is helpful to the HPC administrators since it provides guidance about the real HPC needs. • The data is helpful in better sizing the next HPC generation of supercomputers and to attain a high, steady resource occupation and the best revenue of the funding in terms of scientific results. • Data is useful for system administrators and developers of HPC-related, mostly those involved in developing new algorithms for job scheduling, fault tolerance and minimization of energy consumption, just to mention a few. • This data allow the HPC community to carry out new research on topics such as artificial intelligence methodologies applied to supercomputing, test of resource manager simulators for clusters, etc. • This data are (up to the author's knowledge) the largest publicly available full workload dataset of a supercomputer already structured, making it easier for system users and developers to inspect the data.

## 1 Data

The whole execution trace of Euler cluster during its life is presented as raw data, which can be downloaded from our group's homepage [3], execution traces repository [1] and Mendeley Data [2] in the format of the Parallel Workload Archive [9]. The information contained is quite similar to the one in Euler logs, although curated and anonymized following the Spanish data privacy laws. It comprises 18 fields whose significance is specified in Ref. [1] and replicated here for the sake of clarity and completion. The fields are the following:

1 Job Number (integer): a counter field, starting from 1.
2 Submit Time (in seconds): earliest time the log refers to is zero, and is the submittal time of the first job. The lines in the log are sorted by ascending submittal times, and the jobs are also numbered in this order.
3 Wait Time (in seconds): difference between the job's submit time and the time at which it actually began to run.
4 Run Time (in seconds): wall clock time the job was running (end time minus start time).
5 Number of Allocated Processors (integer).
6 Average CPU Time Used - both user and system (in seconds): average over all processors of the CPU time used, and may therefore be smaller than the wall clock runtime. If a log contains the total CPU time used by all the processors, it is divided by the number of allocated processors to calculate the average.
7 Used Memory -(in Kilobytes): it is again the average per processor.
8 Requested Number of Processors (integer).
9 Requested Time (in seconds).
10 Requested Memory (in Kilobytes per processor).
11 Status 1 if the job was completed; 0 if it failed; and 5 if cancelled.
12 User ID (integer): from one to the number of different users.
13 Group ID (integer): from one to the number of different groups.
14 Executable (integer): from one to the number of different applications appearing in the workload. In cluster Euler this represents a script file used to run jobs rather than the executable directly, so is useless for most cases. It is maintained for compatibility with PWA.
15 Queue Number (integer): from one to the number of different queues in the system. The nature of the queues is specified in an archive header comment.
16 Partition Number (integer): from one to the number of different partitions in the systems. In Euler there is only a partition, so this number is always 1.
17 Preceding Job Number (integer): ID of a previous job in the workload, such that the current job can only start after the termination of this preceding job. Euler does not support chained jobs, so this value is always 0.
18 Think Time from Preceding Job (integer): number of seconds that should elapse between the termination of the preceding job and the submittal of this one. Euler does not support chained jobs, so this value is always 0.

Fields 10, 16, 17 and 18 are unavailable from the PBS stored logs, thus a value of −1 is set throughout these rows according to the PWA format. Additionally, fields 13 and 14 are set to −1 when the owner of the job cannot be identified from the PBS logs and they always correspond to failing jobs. This is due to outages, hangs or reboots of the batch server or allocated nodes without job recovering, which usually imply a forced cleaning.

**Fig. 1:**
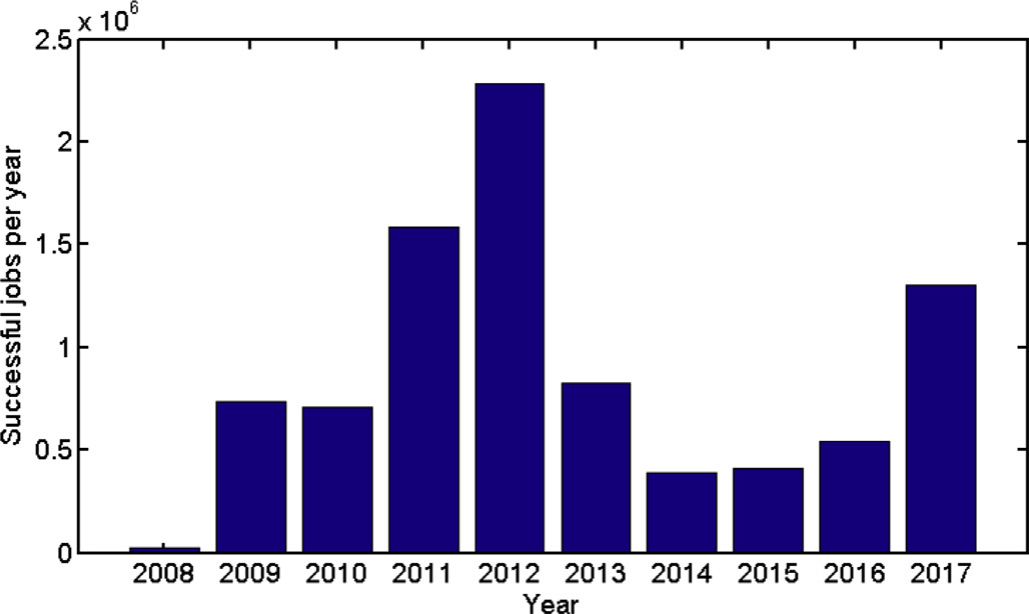
Number of submitted jobs per year during the lifetime of cluster Euler.

**Fig. 2:**
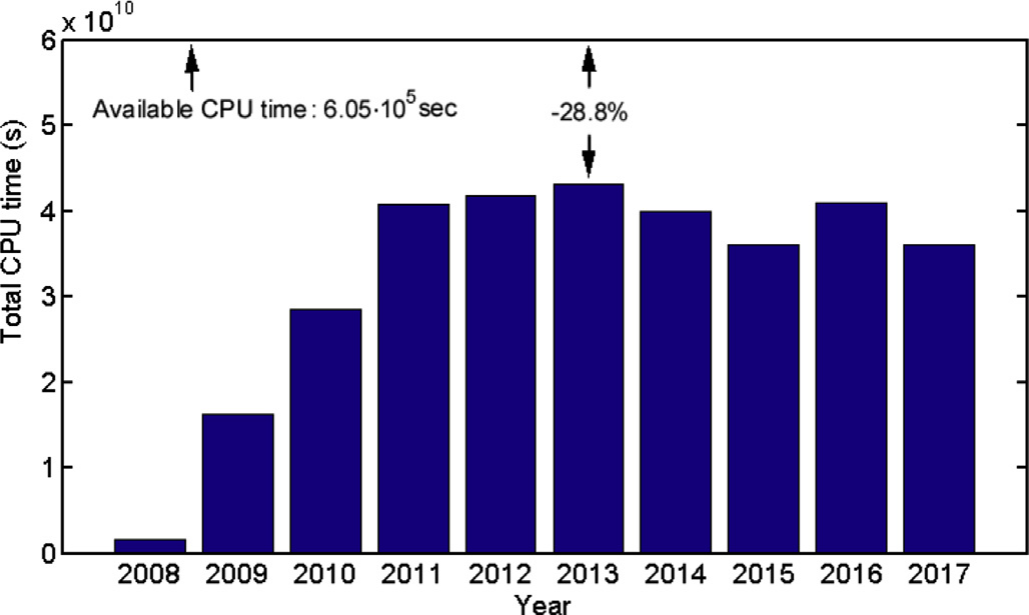
CPU time in seconds consumed by successfully executed jobs per year in cluster Euler. The cluster occupancy is indicated from the distance to the upper border of the plot, which corresponds to the available CPU time per year.

**Fig. 3:**
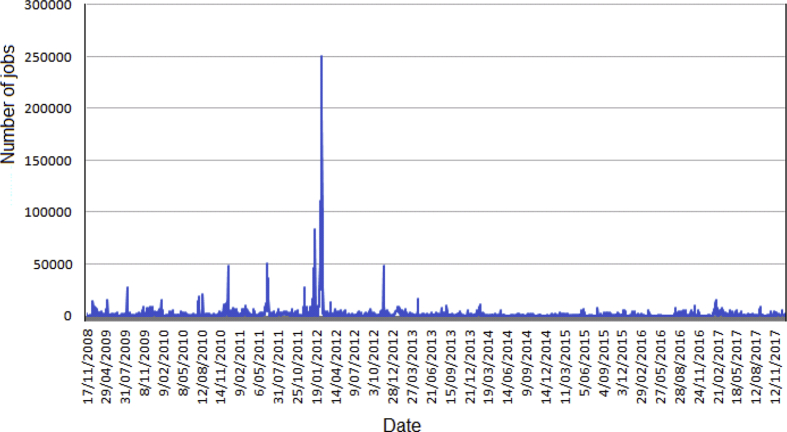
Submitted jobs per day.

**Fig. 4:**
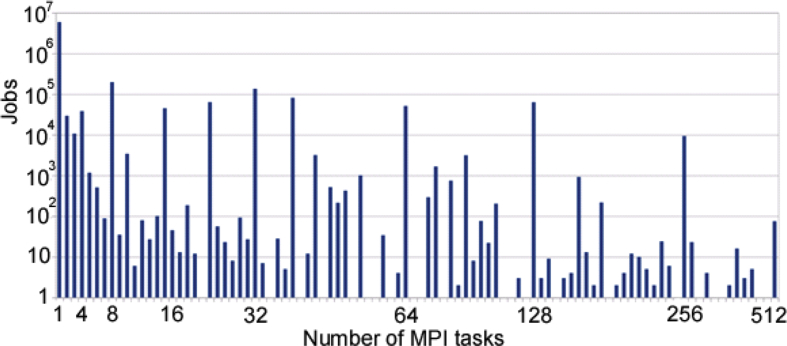
Number of jobs by degree of parallelism.

**Fig. 5:**
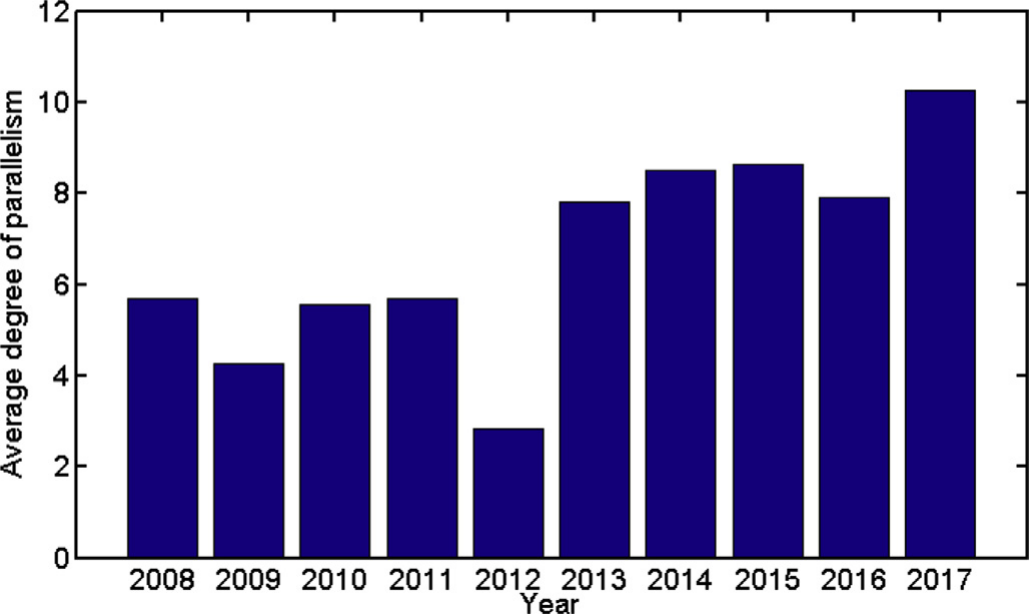
Yearly evolution of the average degree of parallelism in number of cores per job.

**Fig. 6:**
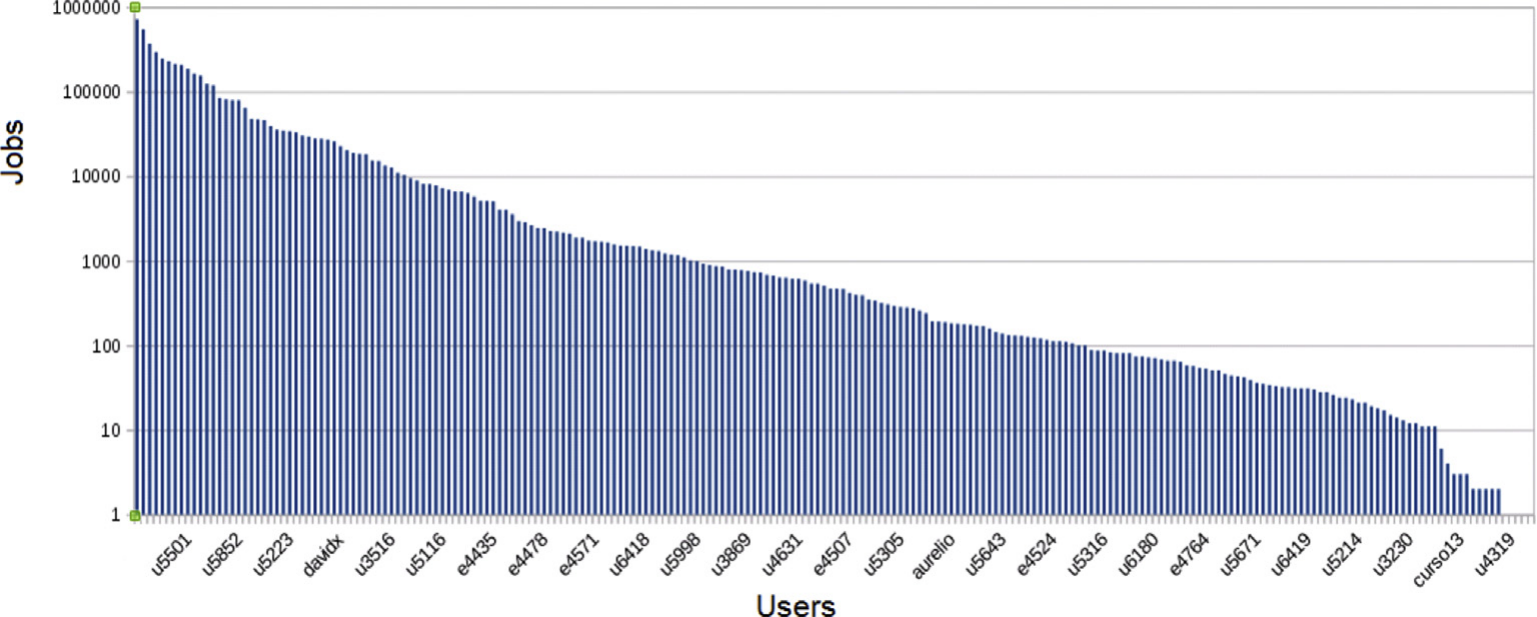
Number of jobs submitted per user.

**Fig. 7:**
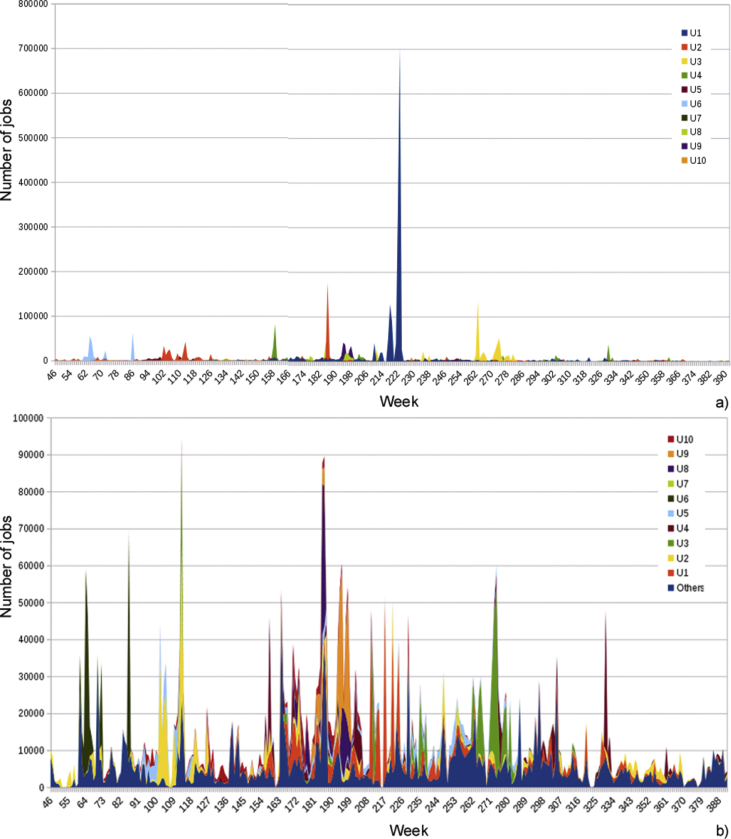
Number of jobs submitted by the a) Most active users along the time; b) Active users, not counting the five most active users, for readability of the usage pattern.

**Fig. 8:**
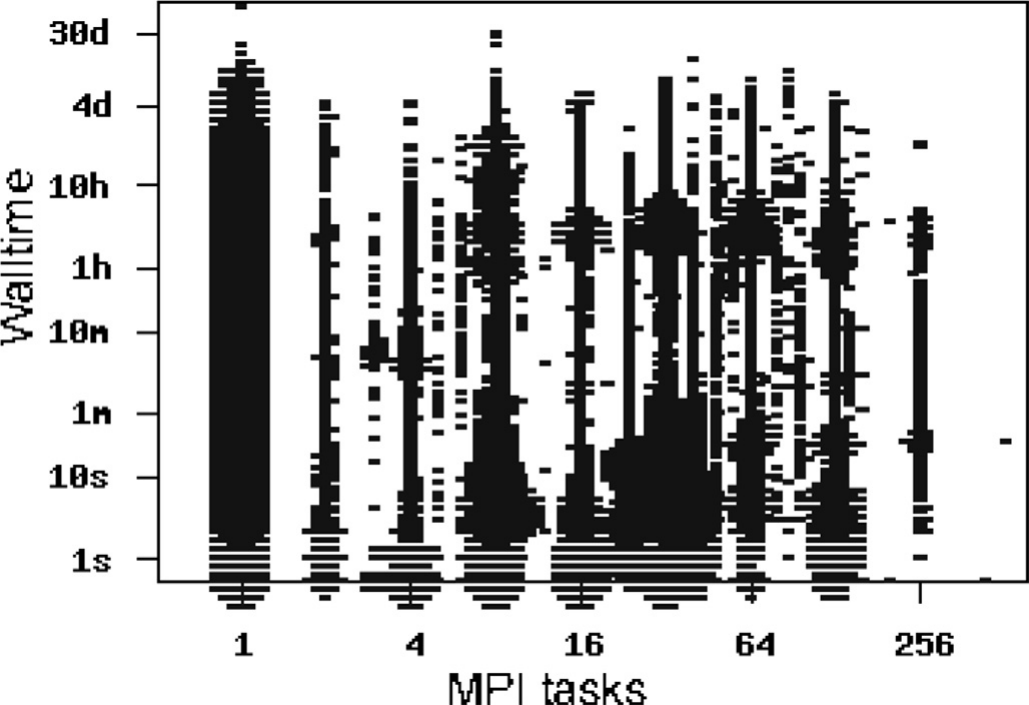
Walltime per job as a function of the degree of parallelization (notation: s - second; m - minute; h - hour; d - day).

**Fig. 9:**
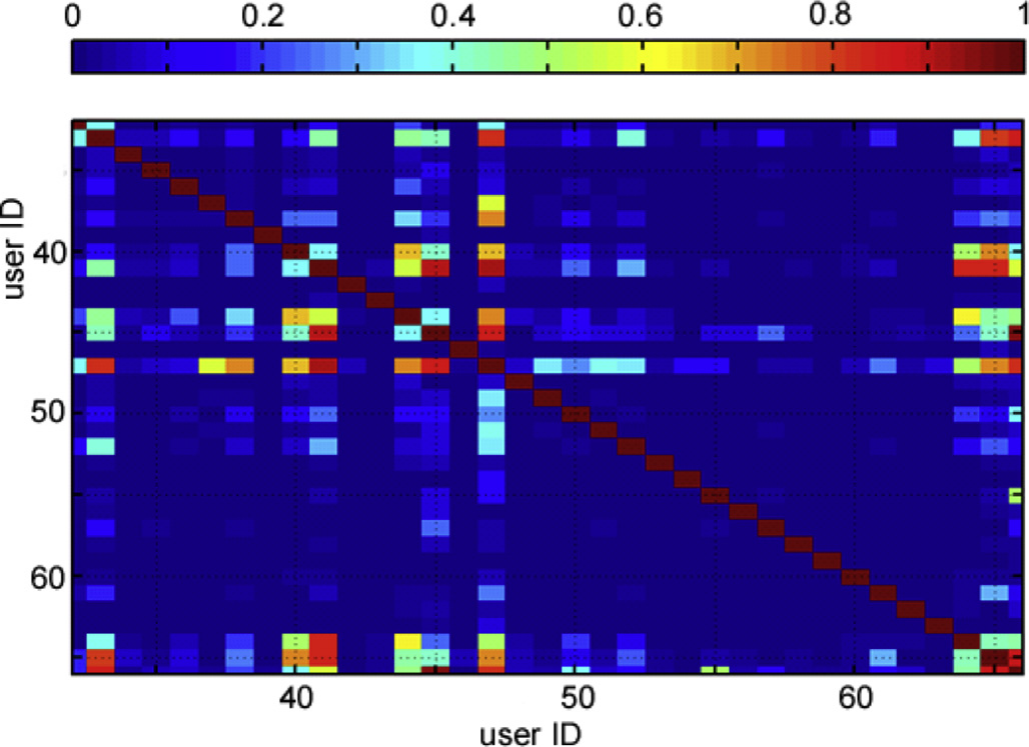
Correlation map based on covariance among user of Euler. Only a portion (39 user IDs of the whole correlation map) is shown.

**Fig. 10:**
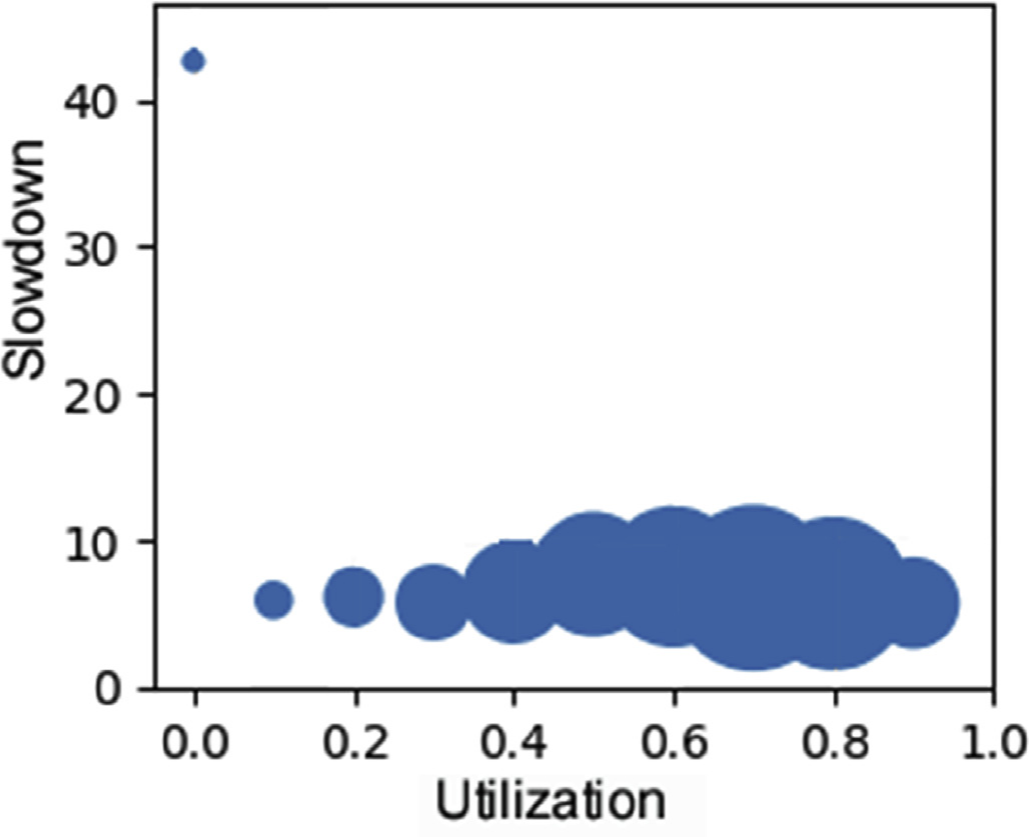
Slowdown as a function of cluster utilization.

Some data statistics are plotted in Fig. 1, Fig. 2, Fig. 3, Fig. 4, Fig. 5, Fig. 6, Fig. 7, Fig. 8, Fig. 9, Fig. 10. Fig. 1 shows the number of submitted jobs per year (submit time is added to the start time given in the description of data collection of the Specifications Table to distribute them per year). Two periods are visible in the plot: a first period with a growing tendency until 2012; and a following second period in which the number of jobs per year quickly decreases (cluster obsolescence). Fig. 2 shows the usage of CPU time over the years. Fig. 3 shows that a constant flow of jobs over time takes place in addition with bursts, that deeply affect the cluster occupation (i.e., about 10% of the jobs submitted in 2012 were submitted on a single day).

Euler is a highly parallel machine and Fig. 4 shows that besides its usage for parallel jobs, there is also a wide number of serial jobs executed. Next, Fig. 5 represents the average degree of parallelism over the years. There is a growing tendency of the degree of parallelism, driven by the increased maturity of the users and the adequacy of their codes to Euler capabilities.

The accumulative job submission by all the users except the 10 top ones has been included in Fig. 6 Besides, the number of jobs submitted by the most active users along the time is included in Fig. 7 (to increase legibility, in Fig. 7b the top 5 values have been removed). Both figures show that a small number of CIEMAT users employ the vast majority of resources: 150 users have submitted over 100 jobs, 90 users over 1000, and only 44 over 10,000 jobs. In fact, the top 5 users have submitted as many jobs as the rest of users all together using short periods instead of a regular, steady usage of the cluster. It can be seen that there is a constant flow of jobs by a large number of users, but the influence of the most active ones is overwhelming on some particular dates.

Walltime as a function of job size (number of MPI tasks) is shown in Fig. 8. Most of the parallel executions are powers of 2, as expected. The largest degree of parallelization is 512, which corresponds to a few of the total executed jobs (less than 100) over the cluster lifetime. Executions with a degree of parallelization of 256 have been performed on a normal basis.

Among the possibilities of the dataset exploitation, there is the statistical analysis to identify cluster misuses and bad praxis by some users, if any. Specifically, by means of the covariance operator it is feasible to determine those users which have been sharing over time, with a high probability, their cluster accounts to boost their computations (see Fig. 9, which plots a portion of the users, anonymized with IDs). This information is useful to the administrators in order to take actions to enforce the code of conduct regarding the access to shared resources at institutional level. In Fig. 9 it is shown that most users exhibit a very small correlation coefficient signal (deep blue), but for some of them located out of the diagonal, the signal is significant (>0.6), which suggests a coordinated use of the accounts by one of the users.

A measure of the time that user's jobs wait for execution is provided by the slowdown metric, which serves to evaluate job schedulers when overload increases or special situations happens [11]. Slowdown is the job's response time (running plus waiting times) normalized by their running time. Utilization is the clusters' filling rate [12]. Both are built out of combinations of various fields of the dataset. The relationship between them is plotted in Fig. 10. The isolated circle at very low values of utilization corresponds to the initial tests performed during the commissioning of the cluster lifetime.

## 2 Experimental design, materials, and methods

Benchmarking of Euler with the Linpack test has revealed an Rpeak performance of 23 TFlops and Rmax of 19.55 Tflops. These values would have put it about position 100th of the Top500 list in June 2008, its acquisition date. Of course, nowadays Euler would be out of that list, with the most powerful supercomputer (Summit, USA) as that of June 2019 being about 10,000 times faster than it.

Euler cluster employs PBS [4] as the Local Resource Management System. MAUI [8] is employed as the external scheduler for PBS. The system is organized with six queues (3 of them are the production queues) devoted to:

• Queue 1 ‘workq’: to support the cluster tests within the commissioning period (disabled on February 17th^,^ 2009 and deleted on May 27th^,^ 2009).
• Queue 2, 3 & 5: production queues (express, normal and eternal, respectively. Enabled on November 27th^,^ 2009).
• Queue 4 ‘batch’: routing queue, which redirects the user request to a production queue depending on the requested walltime. Enabled on November 27th^,^ 2009
• Queue 6 ‘pruebas’: tests queue, enabled on March 16th^,^ 2009.

The differences among them lie in the available resources. The basic idea is that those jobs with less computational requirements be executed on the queues with higher priority. The scheduling policy is the so-called FairShare [10]. Basically, it prioritizes short jobs over long ones, serial over parallel ones, and the jobs of the less active users over more active ones. The timeframe for this is 24h. The set of Figures has been built by postprocessing the execution trace with Python and Matplotlib [5,6] on the open source database server MariaDB.

For the better interpretation of the dataset, it must be clarified that the community of users of cluster Euler is formed by scientific and technical personnel who has a contractual link with the institution. Exceptionally, some users are external to the institution but have been permitted to access to this HPC resource because they are participants in several CIEMAT projects.

## Conflict of Interest

The authors declare that they have no known competing financial interests or personal relationships that could have appeared to influence the work reported in this paper.
